# Factors associated with smoking and e-cigarette use statuses among individuals with chronic diseases after hurricanes Helene and Milton

**DOI:** 10.18332/tid/203427

**Published:** 2025-05-02

**Authors:** Francis Dalisay, Young-Rock Hong, Haoran Chu, Ramzi G. Salloum

**Affiliations:** 1 College of Journalism and Communications, STEM Translational Communication Center, University of Florida, Gainesville, United States; 2 Department of Family and Preventive Medicine, Emory University School of Medicine, Emory University, Atlanta, United States; 3 Public Relations Department, College of Journalism and Communications, University of Florida, Gainesville, United States; 4 Division of Implementation Science and Health Interventions, Department of Health Outcomes and Biomedical Informatics, College of Medicine, University of Florida, Gainesville, United States

**Keywords:** natural disasters, chronic disease

## Abstract

**INTRODUCTION:**

Natural disasters increase health risks for individuals with chronic diseases and may worsen substance use behaviors as a coping mechanism. The present study examined post-disaster intentions to use and past 30-day use of cigarettes and e-cigarettes and associated factors among individuals with chronic diseases who experienced hurricanes Helene and Milton.

**METHODS:**

We conducted online panel surveys in US Federal Emergency Management Agency (FEMA)-declared disaster counties in Georgia, North Carolina, and South Carolina following hurricane Helene, and in Florida following Helene/Milton (October–November 2024). Study participants (n=418) included adults with self-reported diagnoses of diabetes, heart disease, lung/respiratory disease, or cancer. We employed validated measures of hurricane stressors, psychological distress (depression/anxiety), and climate change anxiety. Multiple logistic regression models were used to examine factors associated with intentions to use and past 30-day use of cigarettes and e-cigarettes.

**RESULTS:**

Hurricane stressors were consistently associated with increased intentions and current use of both cigarettes and e-cigarettes (AORs ranging from 1.21 to 1.36, all p<0.001). Depression/anxiety symptoms were associated with increased post-disaster intentions to use cigarettes (AOR=1.91; 95% CI: 1.38–2.64, p<0.001) and e-cigarettes (AOR=1.66; 95% CI: 1.21–2.28, p<0.01), and past 30-day use of cigarettes (AOR=1.49; 95% CI: 1.10–2.02, p<0.05). Climate change anxiety (AOR=1.41; 95% CI: 1.07–1.85, p<0.05) was associated with increased past 30-day e-cigarette use.

**CONCLUSIONS:**

In addition to hurricane stressors and depression and anxiety symptoms, climate change anxiety appears to be a factor associated with tobacco use, particularly, e-cigarettes. Post-disaster health assessments should incorporate substance use interventions for vulnerable populations with chronic conditions.

## INTRODUCTION

Hurricanes Helene and Milton made sequential landfalls in the Southeastern United States in 2024, marking one of the most devastating hurricane seasons in recent history. Helene pummeled through Florida as a strong Category 4 storm, producing winds of up to 140 miles/h, catastrophic storm surges, and deadly tornados^1^. In Georgia, South Carolina, and North Carolina, Helene brought historic heavy rain, flooding, and mudslides, particularly affecting rural communities. Two weeks after the passage of Helene, hurricane Milton made landfall in Florida as a Category 3 storm. Both hurricanes combined resulted in the deaths of at least 250 people and cost an estimated $300 billion in damages^2,3^. These catastrophic events not only caused immediate physical devastation but also posed significant challenges to public health systems and individual health behaviors.

Past research provides evidence of the association between natural disasters and changes in health behaviors, particularly, the increased use of tobacco products^4-6^. Studies have examined post-disaster tobacco use among the general population^7^, young adults^6^, and ever or former smokers^8^, demonstrating consistent patterns of increased substance use following disasters. However, limited research has focused on analyzing factors associated with post-disaster tobacco use among individuals with chronic diseases, despite their heightened vulnerability, and the unique challenges they confront in maintaining health behaviors and health services access during disaster recovery.

The aforementioned gap in knowledge is concerning as people with chronic diseases are at elevated risk for potential substance use^9^. Indeed, research in the US indicates that people with chronic conditions – namely, diabetes, respiratory conditions, cardiovascular diseases, and certain cancers – have higher prevalences of cigarette smoking and vaping than those without these conditions^10-12^. For example, Loretan et al.^10^ analyzed cross-sectional data from the 2019 National Health Interview Survey (NHIS) and reported that an estimated 35% of young adults (18–44 years), 50% of middle-aged adults (45–64 years), and >20% of older adults (≥65 years) who had at least one chronic disease were also current smokers. Additionally, 20% of adults with chronic obstructive pulmonary disease (COPD) and 25% of adults with coronary heart disease were current smokers, which were higher than the estimated prevalence of smoking (14%) among all US adults^11^. The same study by Loretan et al.^10^ found that current cigarette smoking was reported by 49% of middle-aged and 57.6% of older adults who had COPD, 42.8% of older adults who had diabetes, and 51% of young adults who had two or more chronic diseases. In addition, a US National Cancer Institute report that analyzed NHIS data collected between 2018–2022 showed that the prevalences of current cigarette use among cancer survivors between the ages of 18–44 and 45–64 years were respectively 18.5% and 17.6%, which were higher than the prevalences found for those from the remaining US population (12.5% for those aged 18–44 and 17.6% for those aged 45–64 years)^12^. Regarding the use of e-cigarettes, Erhabor et al^13^. analyzed data from the 2021 Behavioral Risk Factor Surveillance System (BRFSS), and reported that the rates of current e-cigarette use were higher among those with cardiovascular disease (10.7%) than those without (6.8%), higher among those with cancer (9.8%) than those without (6.9%), and higher among those with asthma (8.8%) than those without (6.5%).

The relationship between psychological distress and substance use is particularly complex in post-disaster contexts among individuals with chronic conditions. Depression and anxiety are known risk factors for tobacco use among those with chronic conditions^14^, and these vulnerabilities may be amplified during disaster recovery. Forms of psychological distress are well-documented risk factors associated with increases in substance use after experiencing a disaster^7,15^. Alexander and Ward’s^16^ conceptual model for post-disaster substance use provides a theoretical framework, highlighting psychological distress as a central pathway through which negative hurricane experiences could lead to increased substance use. This model suggests a cascade effect: individuals experiencing stressful events as a result of natural disasters develop psychological distress, which may then lead to maladaptive coping strategies, including substances and tobacco use^16^. Stressful experiences that result from a hurricane include one’s home being damaged or destroyed, losing sentimental possessions, or developing health problems. For those suffering from a chronic disease, this pathway may be further complicated by disruptions in access to healthcare occurring after a hurricane (e.g. waiting too many days for an appointment), creating additional sources of psychological distress^17^.

An emerging dimension in disaster-related psychological distress is climate change anxiety, which researchers hypothesize may be associated with increased substance use^18^. Climate change anxiety represents a distinct form of psychological response to climate change characterized by heightened worrying about the topic, which results in cognitive-emotional and functional impairment^19^. Recent research shows that direct and indirect exposure to hurricanes increases climate change anxiety^20^. Climate researchers have attributed the destructive conditions and intensities of hurricanes Helene and Milton to climate change^21,22^. Though no research has explicitly examined climate change anxiety’s role in post-hurricane tobacco use among those with chronic diseases, there is a critical need to investigate such a possibility, as this population faces compound vulnerabilities from both their health conditions and disaster impacts.

The present study aims to address critical gaps in our understanding of post-disaster substance use through one main objective. We examine hurricane-related stressors, depression, anxiety, and climate anxiety as factors associated with post-hurricane intention to use and 30-day use of conventional cigarettes (henceforth, referred to as cigarettes) and e-cigarettes among individuals with chronic diseases who experienced hurricanes Helene and Milton. Findings from this study could inform the development of targeted interventions aimed at mitigating potential increases in tobacco product use that might occur after hurricanes, among people with chronic diseases.

## METHODS

### Study design and sample

This cross-sectional study utilized online panel surveys targeting residents of counties with FEMA disaster declarations in Georgia, North Carolina, and South Carolina following hurricane Helene, and in Florida following Helene/Milton. We contracted Qualtrics (Provo, UT) to collect the data and administer the survey. Qualtrics is a US-based company widely used by academics to conduct survey research^23^. Quota sampling was used to ensure representation across demographic characteristics, including age and gender. In Georgia, North Carolina, and South Carolina, surveys were administered in October 2024, or one month after hurricane Helene made landfall. In Florida, the survey was administered a month after hurricane Milton made landfall, or November 2024. In total, there were 3329 completion attempts. Respondents who did not consent to taking the survey, sped through the survey, or indicated that they did not live in a county impacted by either of the hurricanes, were excluded. This left a final sample size of 1355. The completion rate was 40%. For the current study, we included only participants (n=418) who self-reported a diagnosis of at least one of the following chronic conditions: diabetes, heart disease, lung/respiratory disease, or cancer. These four chronic conditions were chosen because (as opposed to other conditions) they have been typically studied in the context of research looking into post-disaster health outcomes^24,25^. In addition, the four conditions are likely exacerbated by continued tobacco use^10^. *Post hoc* sensitivity analyses confirm the adequacy of our sample size (n=418) for detecting the observed associations between hurricane stressors and tobacco use (for Cohen’s d<0.2, small effect with 80% power). This study followed the Strengthening the Reporting of Observational Studies in Epidemiology (STROBE) reporting guideline. Description of the STROBE reporting guideline and the accompanying STROBE checklist are explained in von Elm et al.^26^.

### Main outcomes

*Intentions to use cigarettes and e-cigarettes*

Two items were used to measure cigarette use intentions and two items were used to measure e-cigarette intentions. The items, adapted from previous research^27^ included: 1) ‘Do you think you will use a [cigarette or e-cigarette] soon’; and 2) ‘Do you think that in the future you might experiment with using a [cigarette or e-cigarette]?’. Responses were measured on a 5-point scale (1=definitely not, 5=definitely yes). Following the approach used by previous research^28^, we classified participants responding with ‘definitely not’ to both intention items (for cigarettes and e-cigarettes) as not having intentions to use, otherwise, we classified them as having intentions to use.

*Past 30-day cigarette and e-cigarette use*

Participants’ past 30-day cigarette and e-cigarette current use were assessed with the following questions: ‘In the past 30 days (i.e. 1 month), on how many days did you smoke cigarettes?’ and ‘In the past 30 days (i.e. 1 month), on how many days have you used an e-cigarette or similar vaping device?’. Participants rated the frequency of cigarette and e-cigarette use on a 7-point scale (1=0 days, 2=1–2 days, 3=3–5 days, 4=6–9 days, 5=10–19 days, 6=20–29 days, 7=daily). Participants who responded with 0 days were classified as non-users, otherwise, they were classified as current users.

### Independent variables

*Hurricane-related stressors*

We asked participants whether in the past month, they had indirectly or directly experienced hurricane-related stressors as a result of hurricane Helene/Milton. We listed the following 10 stressors: lack of any resource (food, water, shelter, electricity) for over a week; loss of sentimental possessions or pets; self or household member had health problems; financial loss; lost access to any needed medical care since the hurricane; lost access to any regular medications due to the hurricane; experienced any delays or difficulties in getting medical appointments since the hurricane; forced to change healthcare providers due to facility closures or damage; housing situation changed as a result of the hurricane; and experienced any issues with mold contamination or structural damage in your home due to the hurricane. Four of the items were adapted from Lowe et al.^29^ and we created 6 of the remaining items. Responses to all the items for hurricane-related stressors were scored with 1=yes, 0=no.

*Psychological distress*

Anxiety

We used four items from the four items from the Generalized Anxiety Disorder-7 (GAD-7)^30^ to measure symptoms of anxiety [e.g. ‘Within the last 2 weeks (14 days), how often have been bothered by feeling nervous, anxious, or on edge?’]. Responses were measured on a 4-point scale (1=not at all, 4=nearly every day). The original GAD-7 comprises 7 items and the scale has been extensively used and validated in people with chronic diseases, such as those examined in the present study^31,32^. For reference, the threshold for determining whether someone is experiencing mild anxiety is a score between 5–9 on the original 7-item scale, moderate anxiety is a score between 10–14, and severe anxiety is a score between 15–21. The original GAD-7 is scored between 0=not at all, to 3=nearly every day.

Depression

We used four items from the Center for Epidemiologic Depression Scale 10 (CES-D 10)^33^ to measure depression symptoms [e.g. ‘Within the last week (7 days), how often have you felt sad?’]. The original CES-D 10 scale comprises 10 items, which has been validated among individuals with chronic disease, such as those examined in our study^34^. For our study, responses were measured on a 4-point scale (1=less than one day, 4=5–7 days). We note for reference that the threshold for determining whether someone is experiencing significant depressive symptoms is a score of 10 on the original full 10-item scale, where each item is scored 0 (less than 1 day) to 3 (5–7 days).

Climate change anxiety

We used three items from Clayton and Karazsia’s^19^ climate anxiety scale to measure this variable (e.g. ‘Thinking about climate change makes it difficult for me to concentrate’). Responses were measured on a 5-point scale (1=strongly disagree, 5=strongly agree).

### Covariates

We measured and controlled for the following covariates that could potentially serve as confounders: age (continuous), biological sex (male, female), state (Florida, Georgia, North Carolina, South Carolina), education level (continuous), income (continuous), and ethnicity (White, non-White Hispanic/Latino, African-American, Asian, or Other). These covariates were selected and adapted from previous research on post-disaster tobacco use conducted by Alexander et al.^8^.

### Statistical analysis

All analyses were undertaken using SPSS version 30^35^. First, we conducted two sets of principal components analyses (PCA). PCA is a dimensionality reduction technique that transforms a set of correlated variables into a smaller number of uncorrelated components, preserving as much variance as possible in the data^36^. This method helps identify underlying structures within the data and is widely used in psychological and social science research to refine measurement scales. The first PCA examined the factor structure and dimensionality for the items measuring hurricane-related stressors, and the second PCA examined the structure and dimensionality of the items measuring psychological distress, i.e. depression, anxiety, and climate anxiety. We used varimax rotation. We retained factors based on inspecting scree plots, examining eigenvalues that were >1, and checking for assumptions of the PCA, namely, strong correlations among features. Second, we used multiple logistic regression models to analyze the associations between the abovementioned independent variables and intentions to use and use of cigarettes and e-cigarettes. With each of the independent variables operationalized using a continuous score, where higher scores indicated greater exposure to stressors or higher levels of psychological distress, we estimated the change in odds associated with a one-point increase in each predictor, while controlling for the abovementioned covariates as potential confounders. Results for the logistic regression models are reported as adjusted odds ratio (AOR) and 95% CI. All tests were two-tailed and the level of statistical significance was p<0.05. In all analyses, listwise deletion was used to deal with missing data, although we should note that the range of missingness was low, between 0.07–0.4%.

## RESULTS

### Sample characteristics

The mean age of participants was 51.2 years (SD=16.97). Other participant characteristics are reported in Tables 1 and 2. The majority of the sample resided in Florida (60.8%), and most identified as White (67%). There were also slightly more males (51.4%) than females (48.1%). Additionally, about 58% of the sample identified as being diagnosed with diabetes, with 36.6% having a diabetes-only diagnosis, while 21.6% identified as being diagnosed with diabetes along with at least one other chronic disease. More than 27% of the sample identified as being comorbid or being diagnosed with two or more chronic diseases.

**Table 1:** Characteristics of study participants with chronic diseases and group comparisons of intentions to use and past 30-day use of cigarettes and e-cigarettes after hurricanes Helene and Milton, cross-sectional survey results (N=418)

*Characteristics*	*Total*	*Intentions to use* *cigarettes*		*Intentions to use* *e-cigarettes*		*Past 30-day use of* *cigarettes*		*Past 30-day use of* *e-cigarettes*	
		*Yes*	*No*	*Yes*	*No*	*Yes*	*No*	*Yes*	*No*
	*n (%)*	*n (%)*		*n (%)*		*n (%)*		*n (%)*	
**State**									
Florida	254 (60.8)	137 (32.8)	117 (28)	132 (31.6)	122 (29.2)	134 (32.1)	120 (28.7)	107 (25.6)	147 (35.2)
Georgia	43 (10.3)	24 (5.7)	19 (4.5)	25 (6.0)	18 (4.3)	22 (5.3)	21 (5.0)	21 (5.0)	22 (5.3)
North Carolina	69 (16.5)	44 (10.5)	25 (6.0)	44 (10.5)	25 (6.0)	35 (8.4)	34 (8.1)	32 (7.7)	37 (8.9)
South Carolina	52 (12.4)	26 (6.2)	26 (6.2)	25 (6.0)	27 (6.5)	22 (5.3)	30 (7.2)	19 (4.5)	33 (7.9)
χ^2^ (p)		2.79 (>0.05)		4.10 (>0.05)		1.89 (>0.05)		1.88 (>0.05)	
**Education level**									
High school or GED	125 (30.0)	68 (16.3)	57 (13.6)	63 (15.1)	62 (14.8)	62 (14.8)	63 (15.1)	55 (13.2)	70 (16.7)
Some college, but less than one year of college credit	38 (9.1)	22 (5.3)	16 (3.8)	24 (5.7)	14 (3.3)	17 (4.1)	21 (5.0)	17 (4.1)	21 (5.0)
>1 year of college, no degree	66 (15.8)	32 (7.7)	34 (8.1)	32 (7.7)	34 (8.1)	32 (7.7)	34 (8.1)	18 (4.3)	48 (11.5)
Associate’s degree	55 (13.2)	30 (7.2)	25 (6.0)	32 (7.7)	23 (5.5)	26 (6.2)	29 (6.9)	25 (6.0)	30 (7.2)
Bachelor’s degree	81 (19.4)	43 (10.3)	38 (9.1)	39 (9.3)	42 (10.0)	38 (9.1)	43 (10.3)	29 (6.9)	52 (12.4)
Master’s degree	31 (7.4)	25 (6.0)	6 (1.4)	24 (5.7)	7 (1.7)	25 (6.0)	6 (1.4)	24 (5.7)	7 (1.7)
Professional degree beyond a Bachelor’s (e.g. MD, DDS, DVM, LLB, JD)	15 (3.6)	7 (1.7)	8 (1.9)	7 (1.7)	8 (1.9)	9 (2.2)	6 (1.4)	8 (1.9)	7 (1.7)
Doctorate degree (e.g. PhD, EdD)	7 (1.7)	4 (1.0)	3 (0.7)	2 (0.5)	5 (1.2)	4 (1.0)	3 (0.7)	3 (0.7)	4 (1.0)
χ^2^ (p)		10.07 (>0.05)		12.8 (>0.05)		13.2 (>0.05)		24.26 (<0.01)	
**Household income in 2023** ($)									
≤19999	56 (13.5)	33 (8.0)	23 (5.5)	30 (7.2)	26 (6.3)	26 (6.3)	30 (7.2)	21 (5.1)	35 (8.5)
20000–39999	122 (29.2)	67 (16.2)	55 (13.3)	53 (12.8)	69 (16.7)	63 (15.2)	59 (14.3)	45 (10.9)	77 (18.6)
40000–59999	74 (17.6)	27 (6.5)	45 (10.9)	31 (7.5)	41 (9.9)	23 (5.6)	49 (11.8)	19 (4.6)	53 (12.8)
60000–79999	52 (12.4)	29 (7.0)	23 (5.6)	29 (7.0)	23 (5.6)	25 (6.0)	27 (6.5)	23 (5.6)	29 (7.0)
80000–99999	31 (7.4)	18 (4.3)	13 (3.1)	20 (4.8)	11 (2.7)	16 (3.9)	15 (3.6)	13 (3.1)	18 (4.3)
100000–119999	19 (4.6)	14 (3.4)	5 (1.2)	15 (3.6)	4 (1.0)	14 (3.4)	5 (1.2)	14 (3.4)	5 (1.2)
120000–139999	13 (3.1)	6 (1.4)	7 (1.7)	8 (1.9)	5 (1.2)	6 (1.4)	7 (1.7)	7 (1.7)	6 (1.4)
140000–159999	19 (4.6)	11 (2.7)	8 (1.9)	11 (2.7)	8 (1.9)	11 (2.7)	8 (1.9)	12 (2.9)	7 (1.7)
160000–169999	12 (2.9)	10 (2.4)	2 (0.5)	11 (2.7)	1 (0.2)	10 (2.4)	2 (0.5)	9 (2.2)	3 (0.7)
170000–189999	9 (2.2)	9 (2.2)	0 (0)	9 (2.2)	0 (0)	7 (1.7)	2 (0.5)	9 (2.2)	0 (0)
≥190000	9 (2.2)	5 (1.2)	4 (1.0)	6 (1.4)	3 (0.7)	5 (1.2)	4 (1.0)	6 (1.4)	3 (0.7)
χ^2^ (p)		23.82 (<0.01)		30.66 (<0.001)		22.87 (<0.05)		40.78 (<0.001)	
**Biological sex**									
Female	197 (47.8)	96 (23.3)	101 (24.5)	92 (22.3)	105 (25.5)	84 (20.4)	113 (27.4)	69 (16.7)	128 (31.1)
Male	215 (51.2)	129 (31.3)	86 (20.9)	129 (31.3)	86 (20.9)	123 (29.9)	92 (22.3)	105 (25.5)	110 (26.7)
χ^2^ (p)		23.82 (<0.05)		7.31 (<0.01)		8.73 (<0.01)		8.04 (<0.01)	
**Race**									
White	277 (66.6)	144 (34.6)	133 (32.0)	137 (32.9)	140 (33.7)	128 (30.8)	149 (35.8)	106 (25.5)	171 (41.1)
Non-white Hispanic/Latino	21 (5.0)	13 (3.1)	8 (1.9)	15 (3.6)	6 (1.4)	11 (2.6)	10 (2.4)	11 (2.6)	10 (2.4)
Black/African American	73 (17.3)	40 (9.6)	33 (7.9)	41 (9.9)	32 (7.7)	42 (10.1)	31 (7.5)	37 (8.9)	36 (8.7)
Asian	4 (0.9)	2 (0.5)	2 (0.5)	2 (0.5)	2 (0.5)	2 (0.5)	2 (0.5)	2 (0.5)	2 (0.5)
Other/mixed race	41 (9.7)	31 (7.5)	10 (2.4)	30 (7.2)	11 (2.6)	29 (7)	12 (2.9)	22 (5.3)	19 (4.6)
χ^2^ (p)		8.50 (>0.05)		11.10 (<0.05)		10.20 (<0.05)		7.03 (>0.05)	
**Chronic disease diagnosis**									
Diabetes-only	155 (37.0)	89 (21.3)	66 (21.3)	92 (22.0)	63 (15.1)	84 (20.1)	71 (17.0)	73 (17.5)	82 (19.6)
Heart disease-only	52 (12.4)	33 (7.9)	19 (4.5)	32 (7.7)	20 (4.8)	29 (6.9)	23 (5.5)	25 (6.0)	27 (6.5)
Lung/respiratory disease-only	59 (14.1)	31 (7.4)	28 (6.7)	29 (6.9)	30 (7.2)	29 (6.9)	30 (7.2)	22 (5.3)	37 (8.9)
Cancer-only	40 (9.6)	18 (4.3)	22 (5.3)	17 (4.1)	23 (5.5)	17 (4.1)	23 (5.5)	13 (3.1)	27 (6.5)
Diabetes and heart disease	31 (7.4)	12 (2.9)	19 (4.5)	9 (2.2)	22 (5.3)	9 (2.2)	22 (5.3)	7 (1.7)	24 (5.7)
Diabetes and lung/respiratory disease	21 (5.0)	14 (3.3)	7 (1.7)	13 (3.1)	8 (1.9)	9 (2.2)	12 (2.9)	12 (2.9)	9 (2.2)
Diabetes and cancer	14 (3.4)	7 (1.7)	7 (1.7)	6 (1.4)	8 (1.9)	7 (1.7)	7 (1.7)	5 (1.2)	9 (2.2)
Heart disease and cancer	15 (3.6)	8 (1.9)	7 (1.7)	8 (1.9)	7 (1.7)	8 (1.9)	7 (1.7)	5 (1.2)	10 (2.4)
Lung/respiratory disease and cancer	5 (1.2)	2 (0.5)	3 (0.7)	3 (0.7)	2 (0.5)	2 (0.5)	3 (0.7)	2 (0.5)	3 (0.7)
Diabetes, heart disease, and cancer	7 (1.7)	3 (0.7)	4 (1.0)	2 (0.5)	5 (1.2)	3 (0.7)	4 (1.0)	2 (0.5)	5 (1.2)
Diabetes, heart disease, and lung/respiratory disease	8 (1.9)	6 (1.4)	2 (0.5)	7 (1.7)	1 (0.2)	7 (1.7)	1 (0.2)	5 (1.2)	3 (0.7)
Diabetes, lung/respiratory disease, and cancer	4 (0.9)	3 (0.7)	1 (0.2)	4 (1)	0 (0)	3 (0.7)	1 (0.2)	3 (0.7)	1 (0.2)
Heart disease, lung/respiratory disease, and cancer	2 (0.5)	2 (0.5)	0 (0)	1 (0.2)	1 (0.2)	2 (0.5)	0 (0)	1 (0.2)	1 (0.2)
Diabetes, heart disease, lung/respiratory disease, and cancer	5 (1.2)	3 (0.7)	2 (0.5)	3 (0.7)	2 (0.5)	4 (1.0)	1 (0.2)	4 (1.0)	1 (0.2)
χ^2^ (p)		12.77 (>0.05)		23.68 (<0.05)		18.14 (>0.05)		18.43 (>0.05)	

**Table 2:** Intentions to use and past 30-day use of cigarettes and e-cigarettes among participants with chronic diseases after hurricanes Helene and Milton, cross-sectional survey results (N=418)

*Intention and usage*	*Total sample* *n (%)*
**Intentions to use cigarettes**	
Yes	231 (55.3)
No	187 (44.7)
**Intentions to use e-cigarettes**	
Yes	226 (54.1)
No	192 (45.9)
**Cigarette use** (past 30 days)	
Yes	213 (51.0)
No	205 (49.0)
**E-cigarette use** (past 30 days)	
Yes	179 (42.8)
No	239 (57.2)

Table 1 also shows results of χ^2^ tests for comparisons of intentions to use and past 30-day use of cigarettes and e-cigarettes between groups. Of note, there were no differences in intentions to use and past 30-day use of cigarettes and e-cigarettes between states. There were, however, differences based on biological sex and income. Table 2 shows descriptive results for intentions to use and past 30-day use of cigarettes and e-cigarettes among our sample of individuals with chronic disease, indicating that about 55% were classified as having the intention to use cigarettes and about 54% were classified as having the intention to use e-cigarettes; 51% of the sample reported past 30-day use of cigarettes, while roughly 43% of the sample reported using e-cigarettes within the past 30 days.

### Principal components analysis (PCA)

The results of the first PCA showed that the items measuring hurricane stressors loaded along a single factor that explained 45.57% of the variance. We summed and combined the items to form a single measure of hurricane stressors (mean=3.86, SD=3.15, α=0.87). The results of the second PCA indicated that the items measuring depression and anxiety loaded along one factor (explaining 54.41% of the variance) and the items measuring climate anxiety loaded along a second factor (explaining 17.39% of the variance). We averaged and combined the items measuring depression and anxiety to form a single measure (mean=2.25, SD=0.90, α=0.94) and the items measuring climate anxiety to form another measure (mean=3.86, SD=3.15, α=0.87).

### Factors associated with post-disaster use and intent to use cigarettes and e-cigarettes

**Figure 1:**
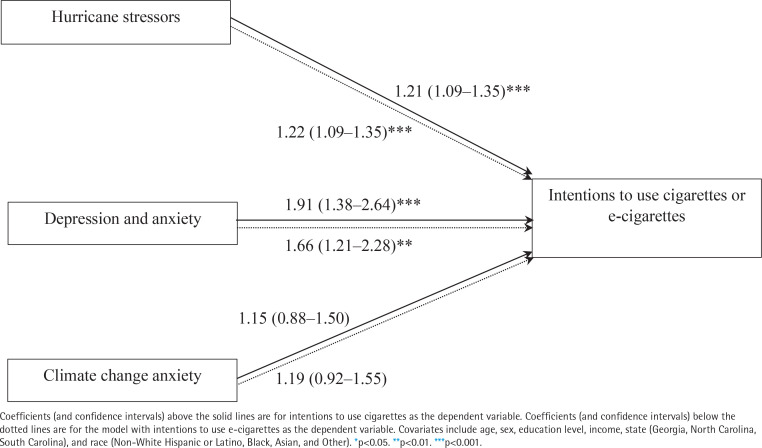
Summary of factors associated with intentions to use cigarettes and e-cigarettes among participants with chronic diseases after hurricanes Helene and Milton, cross-sectional survey results (N=418)

Focusing on the factors we examined, as the results show on Table 3 and Figure 1, after accounting for covariates, hurricane stressors (AOR=1.21; 95% CI: 1.09–1.35, p<0.001) and symptoms of depression/anxiety (AOR=1.91; 95% CI: 1.38–2.64, p<0.001) were associated with increased post-disaster intentions to use cigarettes. Similarly, hurricane stressors (AOR=1.22; 95% CI: 1.09–1.35, p<0.001) and depression/anxiety symptoms (AOR=1.66; 95% CI: 1.21–2.28, p<0.01) were associated with increased intentions to use e-cigarettes, post-disaster. Yet climate change anxiety was not associated with intentions to use either cigarettes or e-cigarettes.

**Table 3:** Factors associated with intentions to use and past 30-day use of combustible cigarettes and e-cigarettes among participants with chronic diseases after hurricanes Helene and Milton, cross-sectional survey results (N=418)

*Factors*	*Intentions to use* *cigarettes*	*Intentions to use* *e-cigarettes*	*Past 30-day* *cigarette use*	*Past 30-day* *e-cigarette use*
	*AOR (95% CI)*			
**Age^a^**	0.96 (0.94–0.97)***	0.96 (0.94–0.98)***	0.97 (0.95–0.99)***	0.96 (0.94-0.98)***
**Sex** (Male=1)	1.65 (1.00–2.72)*	1.92 (1.16–3.17)*	1.91 (1.18–3.07)**	2.05 (0.82–1.12)**
**Education level**	1.01 (0.87–1.17)	0.99 (0.86–1.15)	1.09 (0.94-1.25)	0.96 (0.82–1.12)
**Income**	1.00 (0.89–1.13)	1.15 (1.02-1.30)*	0.99 (0.89–1.11)	1.18 (1.04–1.35)**
**State**				
Georgia	0.38 (0.17–0.85)*	0.58 (0.26–1.30)	0.38 (0.18–0.82)*	0.58 (0.25–1.32)
North Carolina	0.82 (0.39–1.72)	1.14 (0.55–2.33)	0.53 (0.27–1.05)	0.82 (0.39–1.73)
South Carolina	0.53 (0.24–1.15)	0.73 (0.34–1.57)	0.39 (0.18–0.82)*	0.71 (0.31–1.62)
**Race**				
Non-White Hispanic/Latino	0.33 (0.10–1.07)	1.01 (0.30–3.42)	0.36 (0.12–1.14)	0.55 (0.16–1.86)
Black	0.44 (0.22–0.91)*	0.70 (0.35–1.40)	0.88 (0.45–1.71)	0.85 (0.41–1.76)
Asian	0.30 (0.03–2.93)	0.46 (0.04–4.97)	0.61 (0.07–5.79)	0.91 (0.07–12.29)
Other	1.63 (0.64–4.13)	1.47 (0.60–3.62)	1.41 (0.61–3.24)	1.03 (0.43–2.49)
**Hurricane stressors**	1.21 (1.09–1.35)***	1.22 (1.09–1.35)***	1.28 (1.15–1.42)***	1.36 (1.22–1.53)***
**Depression and anxiety**	1.91 (1.38–2.64)***	1.66 (1.21–2.28)**	1.49 (1.10–2.02)*	1.10 (0.78–1.54)
**Climate change anxiety**	1.15 (0.88–1.50)	1.19 (0.92–1.55)	1.13 (0.88–1.46)	1.41 (1.07–1.85)*

a Covariates include age, sex, education level, income, state (Georgia, North Carolina, South Carolina), and race (Non-White Hispanic or Latino, Black, Asian, and Other). Age, education level, and income level were analyzed as continuous variables in regression models. For state, Florida was treated as the reference group and omitted as a state predictor. We thus included only Georgia, North Carolina, and South Carolina as covariates in our regression models. For race, White was treated as the reference group and omitted as a state predictor. We thus included only Non-White Hispanic or Latino, Black, Asian and other ethnicities as covariates in our regression models. Lastly, the three independent variables of hurricane stressors, depression and anxiety, and climate change anxiety were constructed based on results of principal components analyses. AOR: adjusted odds ratio.

* p<0.05.

** p<0.01.

*** p<0.001.

**Figure 2:**
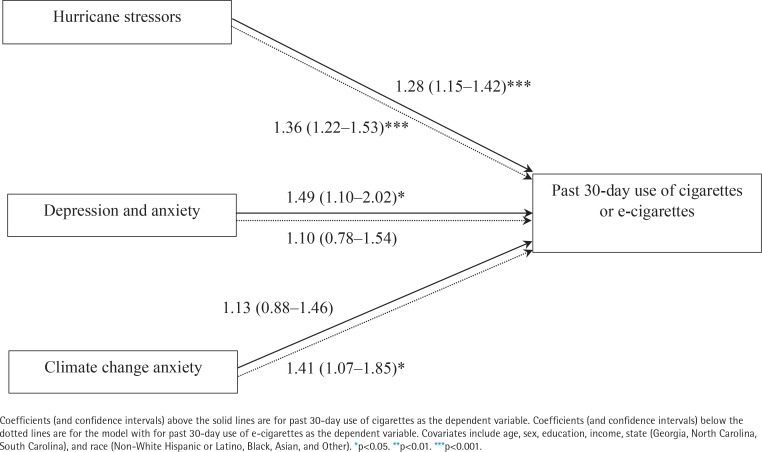
Summary of factors associated with past 30-day use of cigarettes and e-cigarettes among participants with chronic diseases after Hurricanes Helene and Milton, cross-sectional survey results (N=418)

Following the same pattern of the above results for significant predictors of intent to use, hurricane stressors (AOR=1.28; 95% CI: 1.15–1.42, p<0.001) and depression/anxiety symptomology (AOR=1.49; 95% CI: 1.10–2.02, p<0.05) were associated with the increased post-disaster use of cigarettes. Climate change anxiety was not associated with current use of cigarettes (Figure 2).

Regarding factors associated with e-cigarette use, our findings show that hurricane stressors (AOR=1.36; 95% CI: 1.22–1.53, p<0.001) were associated with using e-cigarettes, post-disaster. However, depression/anxiety symptomology was not a significant factor associated with current e-cigarette use. On the other hand, climate change anxiety was associated with increased post-disaster use of e-cigarettes (AOR=1.41; 95% CI: 1.07–1.85, p<0.05).

## DISCUSSION

The present study examined post-disaster intentions to use and past 30-day use of conventional cigarettes and e-cigarettes and associated factors, among individuals with chronic conditions after two major hurricanes in the Southeastern US in 2024. Hurricanes Helene and Milton resulted in hundreds of deaths and billions of dollars in damages^2,3^, creating a context for understanding tobacco use behaviors among vulnerable populations during disaster recovery. Our findings advance the current understanding of post-disaster tobacco use in three key areas.

First, our study identified a high prevalence of post-disaster past 30-day cigarette and e-cigarette use among adults with chronic diseases, i.e. diabetes, heart disease, lung/respiratory disease, or cancer. While our cross-sectional design precluded direct pre-post comparisons, previous research provides evidence that natural disasters could increase tobacco use^5,6^. Yet we also found that cigarettes were being more widely used than e-cigarettes, post-disaster. This finding aligns with recent national survey data indicating that cigarettes remain more commonly used than e-cigarettes^37^. On balance, these rates appear to be higher than those reported in the available literature briefly summarized in the Introduction section^10,12,13^. However, we should reiterate that our sample comprised participants recruited through online panels, and thus, our results may not be completely representative of the counties from where data were collected.

Second, we found that hurricane stressors and symptoms of depression/anxiety were associated with increased post-disaster intentions to use cigarettes and e-cigarettes, and increased past 30-day use of cigarettes. These results support past research that has similarly found that that stressful experiences resulting from a disaster could increase post-disaster use of tobacco^6^ and psychological distress such as anxiety and depression could increase substance use after a disaster^17^. Additionally, although our findings showed that hurricane stressors are associated with increased use of e-cigarettes, the results of our study showed that symptoms of depression/anxiety were not associated with current e-cigarette use. This finding should be interpreted in light of the findings for climate change anxiety, which we discuss below.

Third, we found climate change anxiety is a factor associated with post-hurricane use of e-cigarettes. This finding supports the expectation of researchers, who have proposed that climate change anxiety may lead to increased substance use^18^. Climate change anxiety is a psychological response to climate change characterized by overly worrying about climate change and results in cognitive-emotional and functional impairment^19^. The association with e-cigarettes rather than conventional cigarettes may reflect demographic patterns, as younger individuals tend to both use e-cigarettes more frequently and report higher levels of climate change concern^38-40^. This still warrants further research to analyze the extent to which climate anxiety might increase substance use, more broadly.

Our findings have important implications for healthcare delivery and public health response in disaster-affected regions^41^. Healthcare providers should consider incorporating tobacco use screening into post-disaster health assessments for patients with chronic conditions, particularly when patients report disruptions in their medical care. Such screenings might benefit from including assessments of both disaster-related stress and climate anxiety, given their associations with tobacco use behaviors^42^. Particularly, based on our finding that a factor representing anxiety/depression symptoms was associated with increased intentions to use cigarettes and e-cigarettes, we suggest additional counselling and support for those with anxiety/ depression symptoms to prevent cigarette/e-cigarette uptake. From a public health perspective, disaster response frameworks might benefit from explicitly considering substance use risk among chronically ill populations, ensuring that resource allocation accounts for both the physical and behavioral healthcare needs of this vulnerable population^43^. Future work should focus on developing and evaluating integrated screening protocols that can be feasibly implemented within existing disaster response frameworks while maintaining sensitivity to the unique needs of individuals managing chronic conditions.

### Strengths and limitations

This study’s strengths include its focus on individuals with chronic diseases and comprehensive assessment of multiple psychological factors following major hurricanes. Additionally, our novel incorporation of climate change anxiety as a potential associated factor for post-disaster tobacco use, opens new avenues for understanding emerging psychological influences on health behaviors.

Yet we acknowledge some limitations. First, our study’s one-shot, cross-sectional survey design does not allow for a test of causality. It is equally plausible that the variables we treated as outcomes could serve as antecedents. For instance, there could be a case of reverse causality, where smoking might increase depression/anxiety, rather than depression/anxiety increasing smoking. Furthermore, smoking may increase the prevalence of chronic conditions, rather than chronic conditions predicting smoking. Moreover, our survey also did not assess pre-hurricane smoking status or smoking intentions to see if there was a change. Therefore, we recommend that future research be conducted to test the causal direction between the factors we examined and tobacco use and intent to use. Relatedly, there remain possibilities that cigarette and e-cigarette users may suffer more from the consequences of natural hazards such as hurricanes due to the associated social disadvantages, which may further exacerbate their tobacco use and intent. This therefore necessitates that future research focuses more on the role of disparities in amplifying the potential for tobacco use^44^. Second, our study’s participants were recruited from online panels and were thus not selected through probability-based sampling. Because our sample was not a representative one, our estimates for the prevalences of cigarette and e-cigarette use and intentions may be prone to bias. We, therefore, recommend that future studies use probability-based samples. Third, we examined only a few factors as possible predictors of tobacco use. Past studies have also shown that other factors, such as post-traumatic stress disorder resulting from natural disasters, can impact tobacco use^4,45^. Additionally, the availability and costs of tobacco products might also serve as factors that could affect the chances of using such products in the first place^46^. There may also be residual confounders or additional unknown or unmeasured variables that might be affecting tobacco use status. Thus, we suggest that future studies include a more exhaustive set of potential factors and/or confounders that could be associated with the outcomes we examined. Additionally, we acknowledge the inherent limitations of PCA. While PCA is a useful tool for reducing dimensionality and identifying factor structures, it assumes that components are linearly uncorrelated and may not fully capture the complexity of latent constructs. Furthermore, the choice of rotation and retention criteria can influence factor solutions. Future studies should consider complementary approaches, such as confirmatory factor analysis (CFA), to validate PCA-derived structures. Fourth, another limitation of our study is that it does not explicitly consider the influence of participants’ underlying chronic illnesses on whether or not they took up smoking or vaping. That is, our study did not specifically examine whether the type of chronic illness influences participants’ intention to adopt or exacerbate these behaviors. Arguably, to include such detailed analyses may be beyond the scope of a single study, so we recommend for future studies to examine this potential. Finally, it is possible that there was a selection bias toward a healthy population due to the online recruitment approach used to collect data for our study. That is, presumably, our sample included only those who had access to the internet, and thus, we may have under-sampled those who did not have access to the internet, who may be at a greater disadvantage of experiencing health disparities. Thus, we recommend that future studies include also those who do not have internet access. Relatedly, our findings, since they were based on multiple counties in the Southeastern US, may not be generalizable across other global contexts. Thus, it is important to conduct similar research in different international contexts.

## CONCLUSIONS

Our findings explored and highlighted the complex interplay between disaster experiences, psychological distress, and tobacco use among individuals with chronic conditions. Stressful experiences resulting from a disaster, along with depression and anxiety, could be positively associated with the use of tobacco products among those with chronic diseases. Additionally, climate anxiety could serve as a potential factor associated with the post-disaster use of tobacco, specifically, e-cigarettes. Recently, the Substance Abuse and Mental Health Service Administration^41^ has acknowledged that the prevention and treatment of substance use disorders must be incorporated into disaster preparedness, response, and recovery. Our findings suggest post-disaster health assessments should incorporate substance use – particularly, tobacco use – interventions for populations with chronic health conditions.

## Data Availability

The data supporting this research are available from the authors on reasonable request.
